# Proteomic profiling of plasma exosomes from patients with B-cell acute lymphoblastic leukemia

**DOI:** 10.1038/s41598-022-16282-4

**Published:** 2022-07-13

**Authors:** Shicong Zhu, Cheng Xing, Ruijuan Li, Zhao Cheng, Mingyang Deng, Yunya Luo, Heng Li, Guangsen Zhang, Yue Sheng, Hongling Peng, Zhihua Wang

**Affiliations:** 1 Department of Geriatrics, The Second Xiangya Hospital, Central South University, Changsha, Hunan China; 2 Department of Hematology, The Second Xiangya Hospital, Central South University, Changsha, Hunan China; 3 Institute of Molecular Hematology, Central South University, Changsha, Hunan China

**Keywords:** Cancer, Medical research

## Abstract

We aimed to comprehensively investigate the proteomic profile and underlying biological function of exosomal proteins associated with B-cell acute lymphoblastic leukemia. Exosomes were isolated from plasma samples collected from five patients with B-ALL and five healthy individuals, and their protein content was quantitatively analyzed by liquid chromatography with tandem mass spectrometry. A total of 342 differentially expressed proteins were identified in patients with B-ALL. The DEPs were mainly associated with protein metabolic processes and protein activity regulation and were significantly enriched in the Notch and autophagy pathways. Furthermore, we found that ADAM17 and ATG3 were upregulated in patients with B-ALL and enriched in the Notch and autophagy pathways, respectively. Further western blot analysis of exosomes collected from additional 18 patients with B-ALL and 10 healthy controls confirmed that both ADAM17 and ATG3 were overexpressed in exosomes derived from patients with B-ALL (*p* < 0.001). The areas under the curves of ADAM17 and ATG3 were 0.989 and 0.956, respectively, demonstrating their diagnostic potential. In conclusion, ADAM17 and ATG3 in plasma-derived exosomes may contribute to the progression of B-ALL by regulating the Notch and autophagy pathways. Hence, these proteins may represent valuable diagnostic biomarkers and therapeutic targets for B-ALL.

## Introduction

Acute lymphoblastic leukemia (ALL) is a biologically heterogeneous hematologic malignancy with multiple subtypes characterized by structural chromosomal alterations, followed by somatic alterations and genetic mutations that lead to the dysregulation of cytokine receptors, hematopoietic transcription factors, epigenetic modifiers, and tyrosine kinases^1,2^. Compared with the less-common T-cell precursor lineage, the B-cell precursor lineage subtype is much more prevalent, accounting for approximately 85% of all ALL cases^3^. Despite the dramatic improvements in the overall prognosis of patients with B-cell ALL (B-ALL) promoted by the increased availability of cellular immunotherapy and targeted therapy, the outcome of some B-ALL patients remains poor because of cytokine release syndrome and tumor heterogeneity^4^. Therefore, it is imperative to identify novel biomarkers as effective therapeutic targets for better management of patients with B-ALL.

All cells secrete extracellular vesicles^5^. Exosomes are extracellular vesicles with a size range of 40–200 nm in diameter that originate from endosomes and are released into the extracellular space when multivesicular bodies merge with plasma membranes^6,7^. Based on cell proliferation, extracellular vesicles, including exosomes, contain many components of a cell (such as extracellular matrix, nucleic acids, metabolites, as well as nuclear, cytosolic, and membrane proteins) that are involved in regulating cardiovascular diseases, inflammatory bowel disease, neurodegenerative diseases, and cancers^5,8,9^. In addition, the lipid bilayer maintains the stability of exosomes and protects the proteins and nucleic acids enriched in exosomes from degradation, making exosomes excellent biomarkers and treatment targets for tumors^10^.

Numerous studies have identified exosomal proteins and nucleic acids as biomarkers or therapeutic targets for hematologic malignancies. De Veirman et al. suggested that high expression of miRNA-146a in multiple myeloma-derived exosomes results in the activation of the endogenous Notch pathway, leading to the abnormal release of specific chemokines and cytokines, including interleukin (IL)-6, IL-8, CXCL1, IP-10, and CCL-5, thereby promoting tumor progression^11^. In contrast, inactivation of the Notch pathway by its inhibitor, DAPT, prevents the release of factors regulated by miRNA-146a in multiple myeloma; thus, demonstrating that miRNA-146a can be a treatment target for patients with multiple myeloma^11^. Hun et al. reported that CXCR4, FLT3, and MMP9 in acute myelocytic leukemia-derived exosomes participate in the pathogenesis of acute myelocytic leukemia and can act as potential prognostic biomarkers^12^. Moreover, several exosomal miRNAs, including miR-339-5p, miR-181a, miR-29c-5p, and miR-151-5p, were found to be associated with the survival and development of B-ALL cells; thus, they hold diagnostic and therapeutic potential for B-ALL^13^. Nevertheless, the proteomic profile of B-ALL-derived exosomes and the potential biological functions of exosome proteins in patients with B-ALL need to be elucidated.

Herein, the proteomic profile of plasma-derived exosomes from patients with B-ALL was comprehensively investigated using quantitative proteomic analysis, and the underlying biological function of the identified exosome proteins was evaluated by bioinformatics analyses. Finally, candidate exosome proteins that could be involved in the progression of B-ALL were predicted and validated.

## Results

### Baseline characteristics of B-ALL patients

A total of 18 plasma specimens were collected from patients with primary B-ALL, 11 of which were classified as poor risk group according to the national cancer centre network (NCCN) guidelines, while the remaining 7 were classified as good risk group. Of them, 4 presented EPPK1 mutation, 3 presented PDE4DIP mutation, 3 presented BCR mutation, and 3 presented TP53 mutation. Additionally, 7 patients harbored t(9; 22)(q34; q11), encoding the fusion gene of *BCR-ABL1* (Table 1).

**Table 1:** Patients with B-ALL general information.

Sample	Age/Sex	Type	Clinical status	Primary cytogenetic abnormality	Mutation	Fusion gene	Blasts (%)	Differentiation antigen expression
1	26/F	Plasm	ND	46, XX, t(12;15)(p13;q25)^12^ /46, XX^8^	JAK2, EPPK1, PDE4DIP, FOXP1	ETV6-NTRK3	76%	CD34, CD10, CD19, CD22, HLA-DR, CD38
2	21/F	Plasm	ND	46, XX, t(4; 11)(q21; q22)^8^/46,XX^12^	KMT2C, BCL11B, FANCD2	KMT2A-AFF1	77%	CD34, CD19, CD22, HLA-DR
3	35/M	Plasm	ND	46, XY, inv (1) (p13q21) ^5^/46, XY^1^	DDX41, PMS2, ERCC6L2, SLX4	Missing	94.5%	CD34, CD10, HLA-DR, CD19, CD22, CD20, CD38
4	47/F	Plasm	ND	45, XX, der(3;7)(q10;q10),t(9;22)(q34;q11.2)^3^/45,idem, + 1, dic(1;17)(p13;p11)^2^/46,XX^15^	BRCA2, EFL1, ACD	BCR-ABL1	86%	CD34, CD10, CD19, HLA-DR, CD13
5	35/F	Plasm	ND	46, XX^5^	EPAS1, ZNF479, BCLF1	BCR-ABL1	43%	CD34, CD10, CD19, HLA-DR, CD22
6	19/M	Plasm	ND	45, XY, − 3, add(7)(p11), − 9, del(22)(q11), + mar^12^/46, XY^6^	CTNND2, CHD4, NUP214, EPPK1, NCOR1, BRCA2	Missing	98%	CD34, CD10, CD19, CD22, HLA-DR, CD38
7	46/F	Plasm	ND	46, XX, t(9; 22)(q34; q11.2)^18^/46, XX^2^	RUNX1, BCR, IDH1, ARID4A, RAD50	BCR-ABL1	88%	CD10, CD19, CD38, CD20, CD13, CD33
8	25/M	Plasm	ND	46, XY^8^	HGF, PDE4DIP, TRIP11	Missing	81%	CD34, CD10, CD19, CD22, HLA-DR, CD20, CD38, CD13
9	32/M	Plasm	ND	47, XY, + X, t(4;11)(q21;q23), − 17, + r^17^/46, XY^3^	TP53, FANCD2	KMT2A-AFF1	82.5%	CD34, CD19, CD22, HLA-DR, CD38
10	45/F	Plasm	ND	46, XX, t(9; 22)(q34; q11.2)^20^	PHF6, FAM47C, ARID1B, SUFU	BCR-ABL1	87%	CD34, CD10, CD19, CD22, CD38, HLA-DR, CD20
11	48/M	Plasm	ND	46, XY^17^	TP53, CCND3, ID3, MYC, TP63	Missing	88.5%	CD10, CD19, HLA-DR, CD22, CD20, CD38
12	36/M	Plasm	ND	46, XY^20^	PTEN, MYC, EPPK1, BRINP3	Missing	81%	CD34, CD10, CD19, HLA-DR, CD38
13	45/M	Plasm	ND	Missing	PTEN, RPL22, PRB2, TAF1	Missing	97%	CD34, CD10, CD19, HLA-DR
14	34/F	Plasm	ND	46, XX^14^	Missing	Missing	66%	CD34, CD10, CD19, CD79a, TDT, HLA-DR
15	47/M	Plasm	ND	46, XY, t(9; 22)(q34; q11.2)^4^/45, idem, − 7^16^	GRIN2A, SLX4, AXIN1, PCLO, CDH11	BCR-ABL1	72.5%	CD34, CD10, CD19, CD22, HLA-DR
16	34/F	Plasm	ND	46, XX^20^	TP53, EPPK1, FBLN2, MYD88, IRF4, AURKA	Missing	79%	CD10, CD19, CD22, CD20, CD38, HLA-DR
17	49/M	Plasm	ND	46, XY, t(9; 22)(q34; q11.2)^16^	FOXP2, AMER1, BCR, PDE4DIP, CDC73	BCR-ABL1	89.1%	CD34, CD10, CD19, CD38, HLA-DR
18	55/M	Plasm	ND	46, XY, t(9; 22)(q34; q11)^15^/47,idem, + der(22)t(9; 22)^1^/48, idem, + 8, + ?13^1^/46, XY^3^	ASXL1, EPHA3, BCR	BCR-ABL1	78%	CD34, CD10, CD19, CD22, HLA-DR

*M* Male; *F* Female; *ND* Newly diagnosed.

### Two-step protocol comprising discovery and verification phases were designed

**Figure 1:**
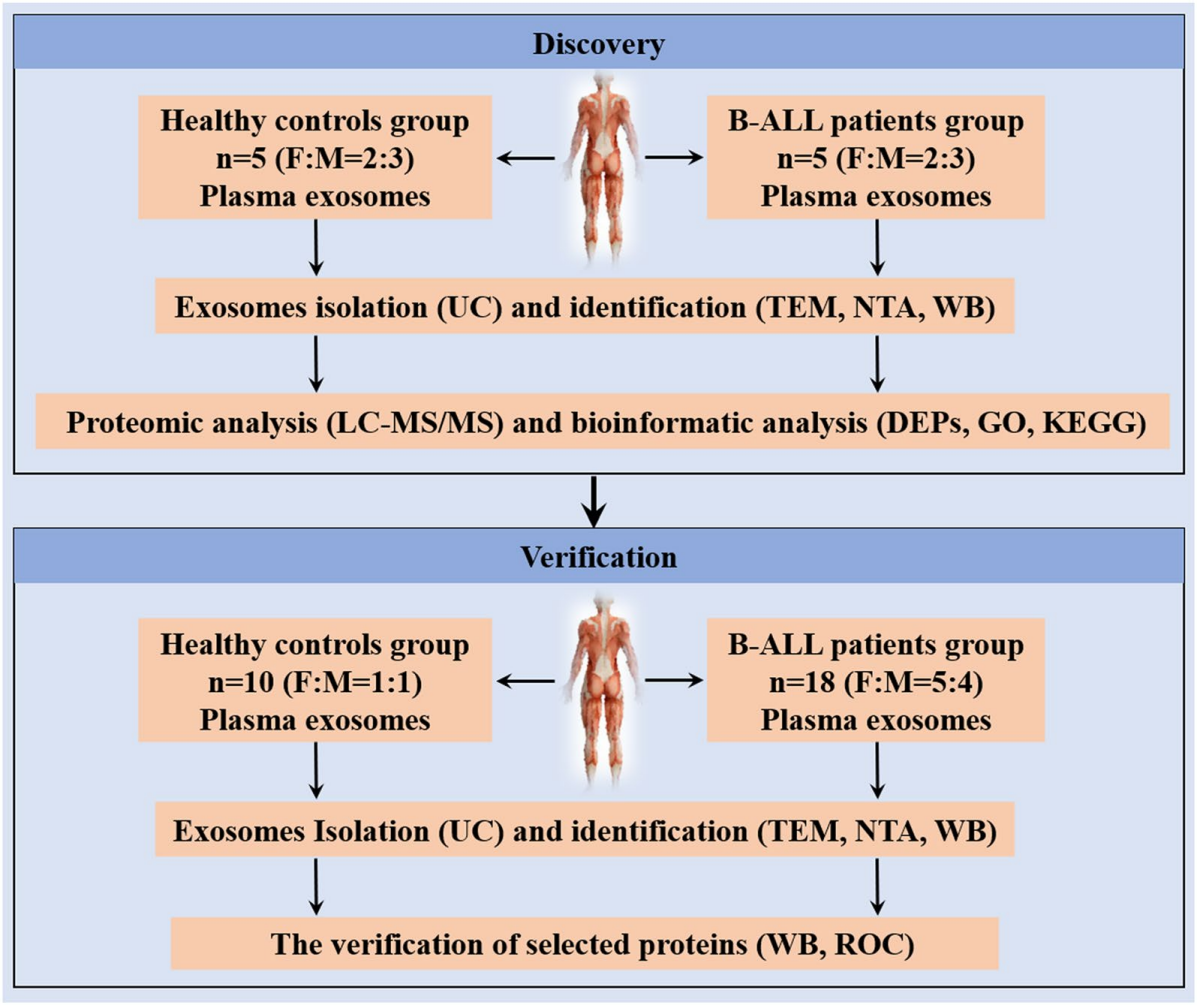
Schematic overview of the study. Discovery phase: prediction of the differentially expressed exosome proteins using proteomic and bioinformatic analyses. Verification phase: verification of selected differentially expressed exosome proteins by WB analysis and ROC curves. B-ALL, B-cell acute lymphoblastic leukemia; UC, ultracentrifugation; TEM, transmission electron microscope; NTA, nanoparticle tracking analysis; WB, western blot; ROC, receiver operating characteristic; F, female, M, male.

To analyze the proteomic profile of plasma-derived exosomes associated with B-ALL, we designed a two-step protocol comprising discovery and verification phases (Fig. 1). In the discovery phase, we extracted plasma exosomes from five patients with B-ALL and five healthy volunteers by ultracentrifugation; the plasma exosomes were then identified by transmission electron microscopy and nanoparticle tracking analysis, and specific antibodies were used to detect TSG101 and CD81 by western blot. To obtain the proteome of plasma exosomes, proteomic analysis was performed by liquid chromatography with tandem mass spectrometry and differentially expressed exosome proteins between patients with B-ALL and healthy individuals were identified. Bioinformatics analyses, including gene ontology (GO) function enrichment analysis and Kyoto Encyclopedia of Genes and Genomes (KEGG) pathway enrichment analysis, were performed on the identified differentially expressed proteins (DEPs). The candidate proteins involved in the progression of B-ALL were predicted based on KEGG pathway enrichment analysis, which were validated in the verification phase. Briefly, plasma exosomes were extracted from 18 patients with B-ALL and 10 healthy controls by ultracentrifugation and were characterized by transmission electron microscopy, nanoparticle tracking analysis, and western blot. Thereafter, the levels of predicted candidate proteins in patients with B-ALL and the healthy population were compared by western blot, and their clinical performance was determined by analysis of their receiver operating characteristic curves.

### Plasma exosomes were successfully extracted from participants using ultracentrifugation

**Figure 2:**
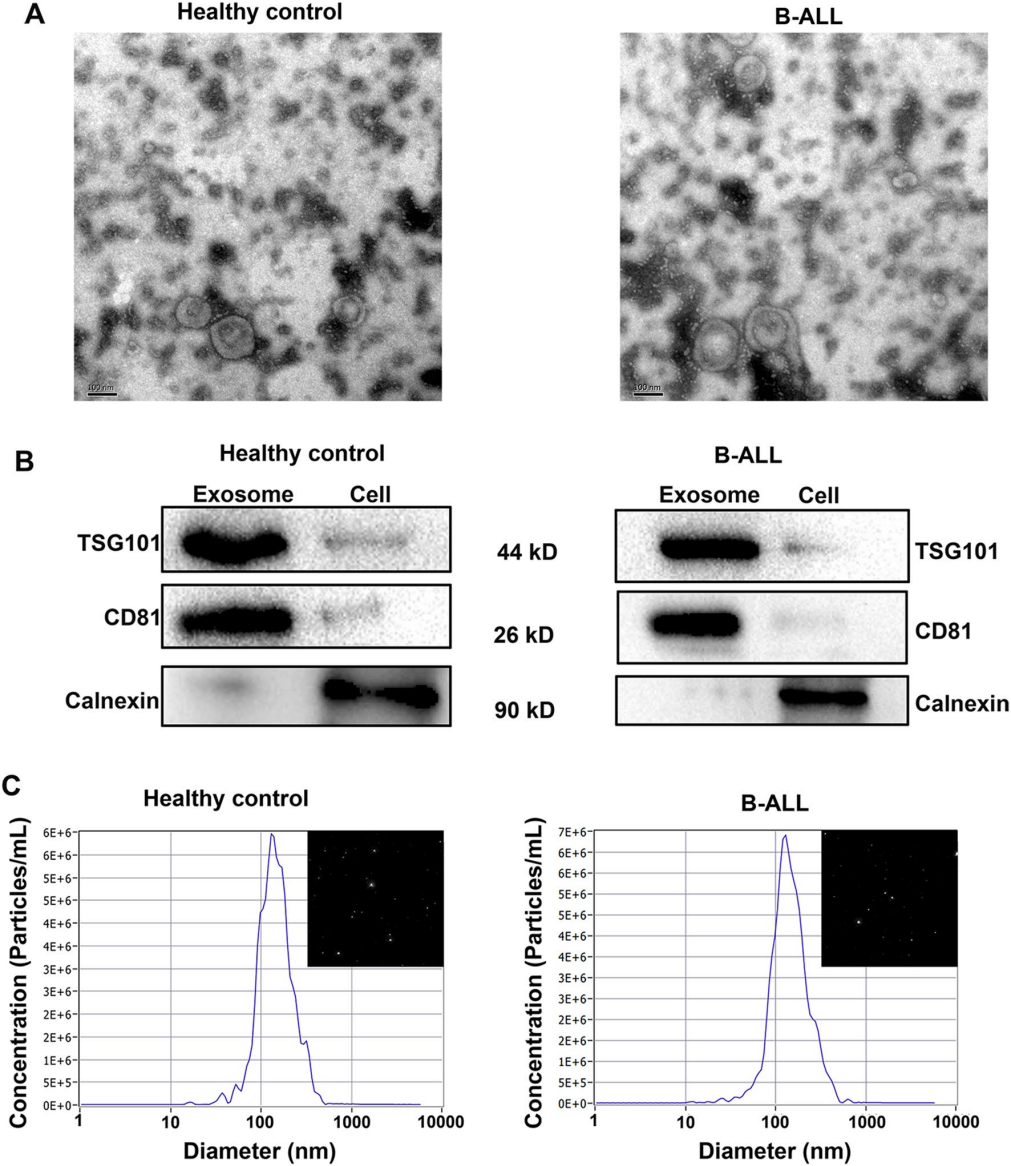
Plasma exosome characterization. (**A**) Representative morphology of the exosomes by transmission electron microscopy. (**B**) Detection of specific surface markers in the isolated exosomes by western blot. (**C**) The size of the isolated exosomes was determined by nanoparticle tracking analysis.

Plasma exosomes were extracted from patients with B-ALL and healthy volunteers by ultracentrifugation, and their morphology was captured by transmission electron microscopy (Fig. 2A). A representative cup shape and spherical vesicles with a size range of approximately 80–150 nm were observed. Western blot analysis suggested that the specific surface proteins of exosomes, including TSG101 and CD81, were present in the isolated vesicles^14^, whereas calnexin, an endoplasmic reticulum-specific protein^15^, was absent (Fig. 2B). Nanoparticle tracking analysis further confirmed that the size of the extracted plasma exosomes ranged from 80 to 200 nm, with a primary peak size of 136 nm in B-ALL group and 138 nm in healthy control group. Additionally, the concentration of exosome extracted from B-ALL patients (1.8 × 10^10^ particles/ml) was higher than that extracted fromhealthy volunteers (1.7 × 10^10^ particles/ml) (Fig. 2C).

### Dfferentloally expressed exosome proteins were existed between patients with B-ALL and healthy individuals

**Figure 3:**
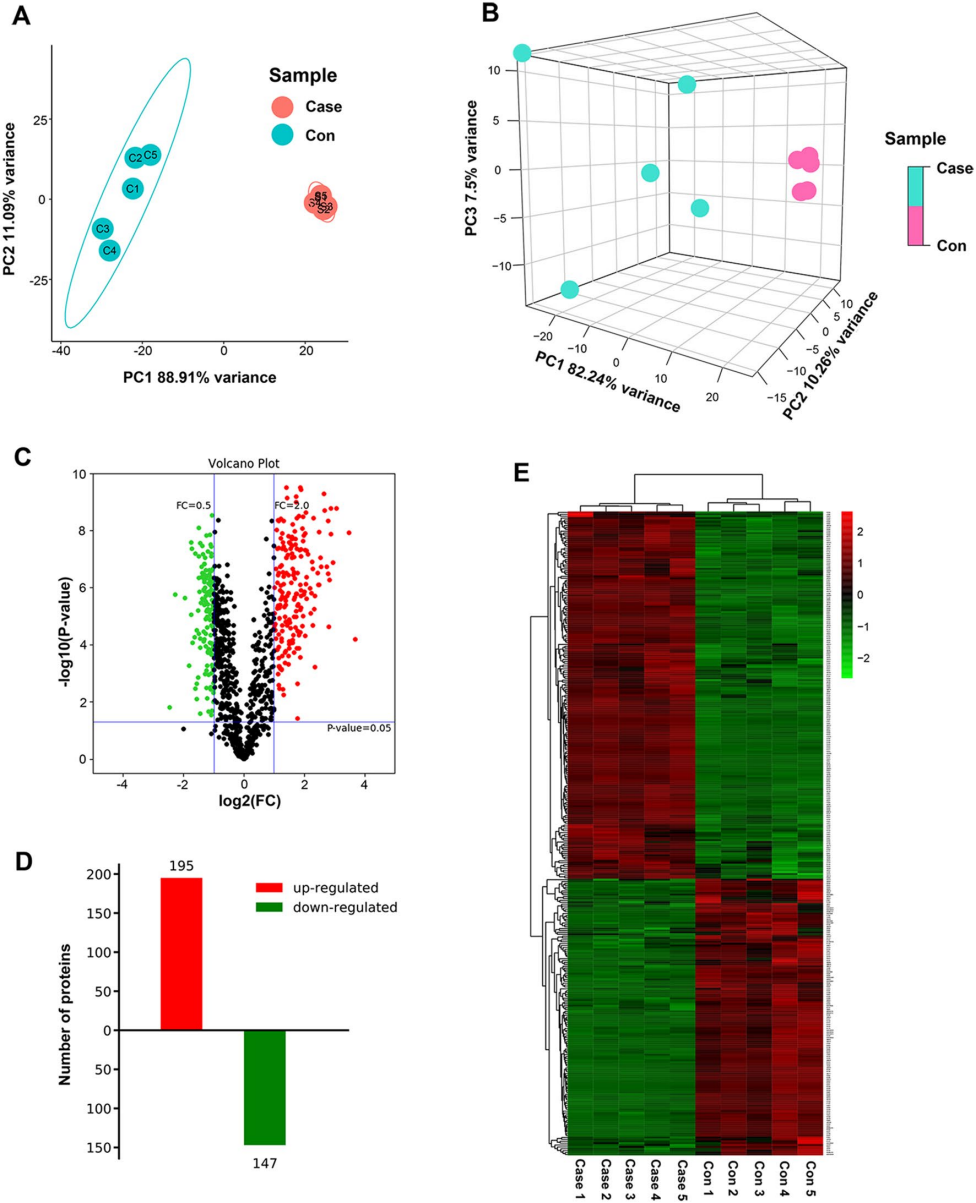
Proteomics analysis of the exosomes. (**A**,**B**) Principal component analysis of the individual samples. (**C**) The volcano plot of the differentially expressed exosome proteins between B-ALL patients and healthy controls. Green and red points represent downregulated and upregulated proteins, respectively. (**D**) Number of upregulated and downregulated differentially expressed exosome proteins in B-ALL patients compared with healthy controls. (**E**) Heatmap clustering of the differentially expressed exosome proteins.

Proteomic analysis of the isolated exosomes was then performed by liquid chromatography with tandem mass spectrometry. Principal component analysis showed that B-ALL patient-derived exosomes had a different protein profile as compared with that of healthy individuals-derived exosomes, as indicated by the significant differences in spatial distribution (Fig. 3A,B). Next, the differentially expressed exosome proteins between patients with B-ALL and healthy individuals were selected based on the criteria of > twofold change or < 0.5-fold change, and *p* < 0.05 (Fig. 3C). A total of 342 DEPs were identified, of which 195 and 147 were upregulated and downregulated proteins, respectively (Fig. 3D). Detailed information on all DEPs is provided in Supplementary Table S1. Heatmap clustering clearly demonstrated a separation within the shortlisted proteins between the B-ALL patient- and healthy individual-derived exosomes, showing different patterns in each group (Fig. 3E).

### The identifited DEPs were significantly enriched in the Notch and autophagy pathways

**Figure 4:**
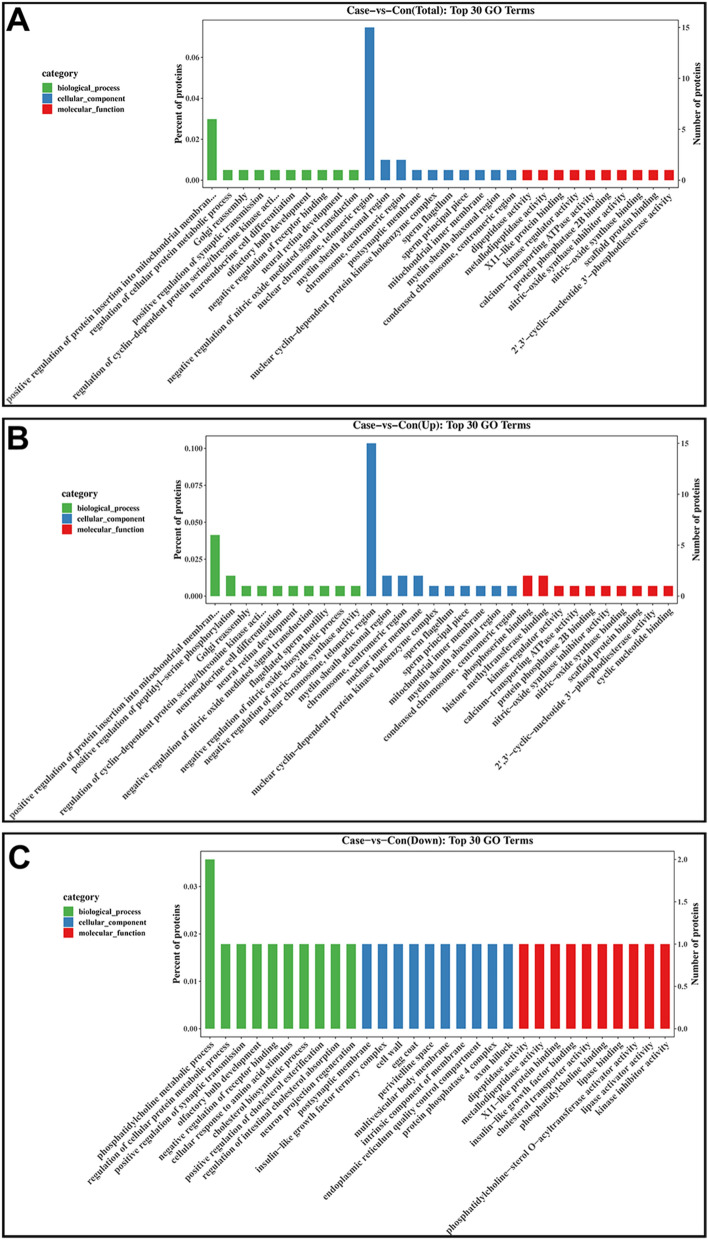
Gene ontology function enrichment analysis of the differentially expressed proteins (DEPs). Top 30 enriched terms of the (**A**) all DEPs, (**B**) upregulated DEPs, and (**C**) downregulated DEPs.

**Figure 5:**
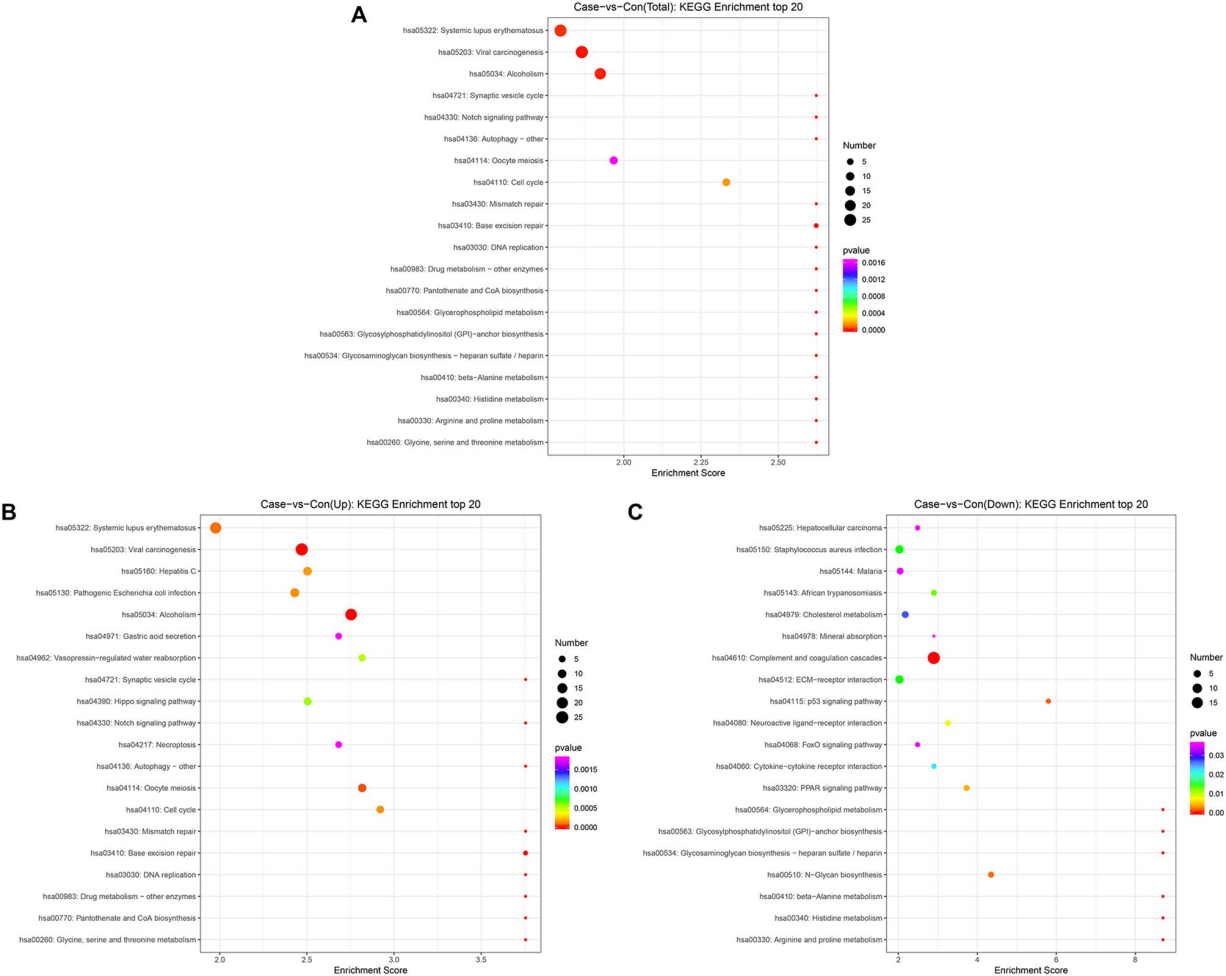
KEGG pathway enrichment analysis of the DEPs. Top 20 enriched pathways of (**A**) all DEPs, (**B**) upregulated DEPs, and (**C**) downregulated DEPs.

GO and KEGG enrichment analyses were performed to understand the respective functions and pathway enrichments of all 342 DEPs. The top 30 GO enrichment terms of all DEPs, upregulated DEPs, and downregulated DEPs are shown in Fig. 4A,B,C, respectively. Detailed information, including all DEPs, is shown in Supplementary Tables S2–4. GO enrichment analysis of the DEPs showed that the terms were distributed into three categories: biological process, cellular component, and molecular function. For the biological process category, all proteins, as well as the upregulated proteins, were significantly enriched in the positive regulation of protein insertion into the mitochondrial membrane involved in the apoptotic signaling pathway, whereas the downregulated proteins were mainly enriched in the phosphatidylcholine metabolic process. For the cellular component category, both total proteins and upregulated proteins were significantly enriched in the nuclear and telomeric regions, whereas downregulated proteins were largely enriched in the postsynaptic membrane. In the molecular function category, both total proteins and downregulated proteins were mainly enriched in dipeptidase activity, whereas the upregulated proteins were significantly enriched in phosphoserine binding. Functional enrichment analysis showed that the DEPs were mainly associated with protein metabolic processes and protein activity regulation. The top 20 KEGG enrichment pathways of all DEPs, upregulated DEPs, and downregulated DEPs are shown in Fig. 5A,B,C, respectively. Details of the DEPs enriched in each pathway are shown in Supplementary Tables S5–7. The results showed that all DEPs were significantly enriched in the Notch and autophagy pathways, which have been previously associated with the occurrence and development of ALL^16,17^.

### ADAM17 and ATG3 were identified as valuable biomarkers of B-ALL

**Figure 6:**
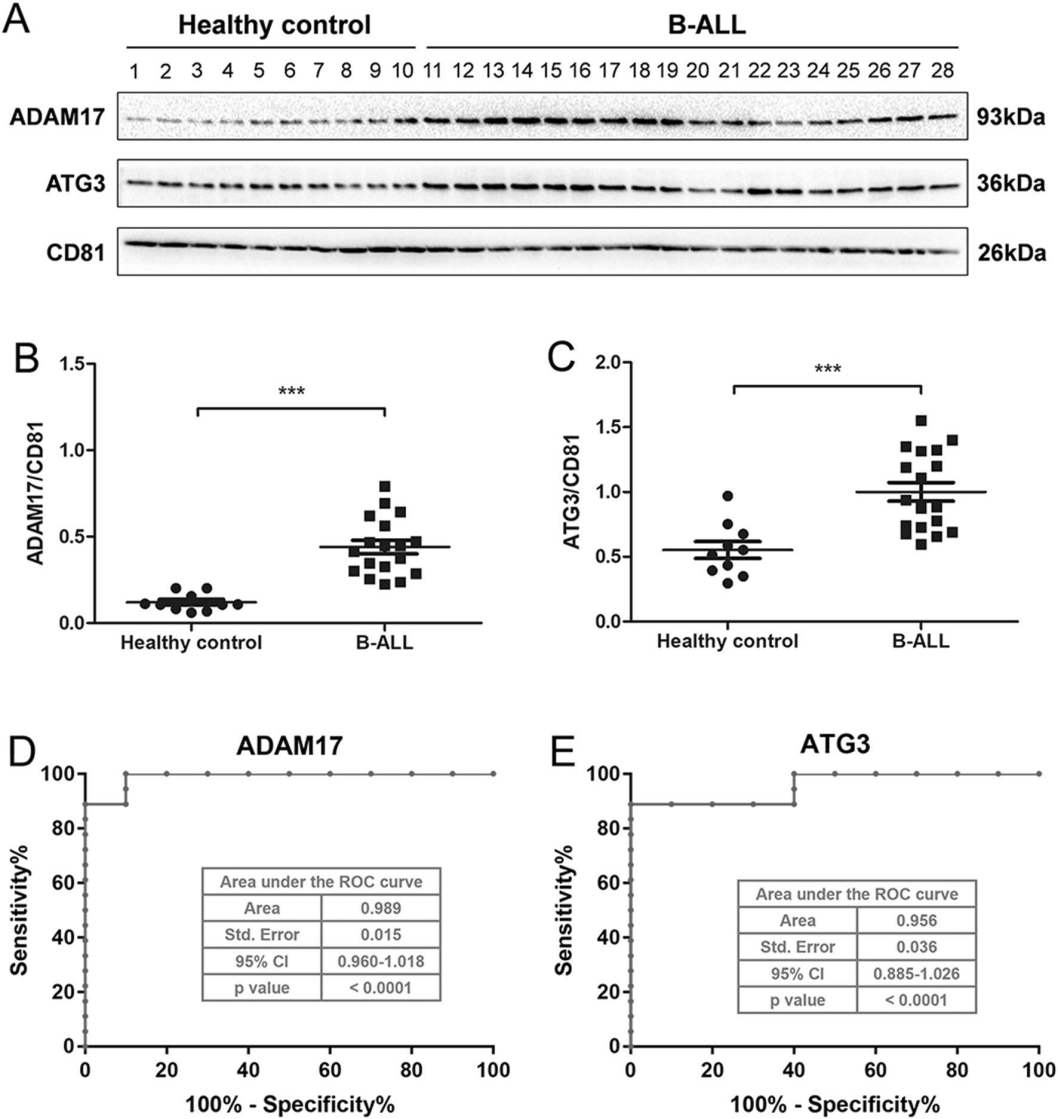
Verification of the predicted candidate proteins. (**A**) Western blot and densitometric analysis of the respective protein band of (**B**) ADAM17 and (**C**) ATG3. The blots were cut prior to hybridization with antibodies. Receiver operating characteristic curves of (**D**) ADAM17 and (**E**) ATG3.

Based on the KEGG enrichment analysis results, the proteins that were enriched in the Notch signaling pathway and autophagy-other pathway were selected as potential proteins involved in B-ALL progression. In particular, ADAM17 was enriched in the Notch signaling pathway, and ATG3 was enriched in the autophagy-other pathway (Supplementary Table S7). Verification experiments revealed that both ADAM17 and ATG3 were highly expressed in B-ALL patient-derived exosomes compared with those derived from healthy controls (Fig. 6A–C), which was consistent with the proteomics analysis (Supplementary Table S7). Furthermore, the clinical utility of ADAM17 and ATG3 was evaluated by analysis of their receiver operating characteristic curves. The results demonstrated that the area under the curve of ADAM17 reached 0.989 and that of ATG3 reached 0.956 (Fig. 6D,E), thereby suggesting that these two proteins may represent valuable biomarkers of B-ALL.

## Discussion

There are two limitations in our current study. On one hand, the sample size in our current study is too small to obtain the exact result, and we will continue to expand the sample size to verify our present study and lucubrate the underlying biological function of exosomal proteins in B-ALL patients. On the other hand, we used an ultracentrifugation methodology to isolated plasma exosomes. A major issue on the isolation of plasma exosomes is the high abundance of serum proteins, such as albumin and globulins, and non-exosome lipid particles such as chylomicrons and lipoprotein particles that can interfere with particle counts. Furthermore, the levels of chylomicrons and lipoprotein can vary greatly from individual to individual and are influenced, among others, by diet and genetics adding further complexity to biomarker validation studies. Therefore, separation of plasma exosomes from soluble proteins and non-exosome lipid particles is critically important in biomarker discovery and validation.

Exosomes have been recently considered as a high-quality liquid biopsy medium for cancer investigations because of their abundant stable, functional proteins and nucleic acids^18^. In the current study, we comprehensively analyzed the protein profiles of exosomes derived from the plasma of patients with B-ALL using proteomic approaches to identify potential exosome proteins as diagnostic biomarkers for patients with B-ALL. Principal component analysis showed clearly distinguishable protein profiles between exosomes derived from patients with B-ALL and those from healthy individuals. Overall, 342 exosome DEPs were identified in patients with B-ALL compared with healthy controls, among which 195 were upregulated, and 147 were downregulated. GO function enrichment analysis suggested that the DEPs were mainly involved in protein metabolic processes and regulation of protein activity. KEGG pathway enrichment analysis also showed that the DEPs were significantly enriched in the Notch and autophagy pathways. The Notch signaling pathway is a functional pathway that controls cell survival, proliferation, differentiation, apoptosis, migration, and invasion, and is involved in the determination of cell biological functions in both normal and pathological tissues^19–21^. Several studies have revealed the regulatory effect of the Notch signaling pathway in multiple cancers, including breast cancer^22^, colorectal cancer^23^, acute myeloid leukemia^24^, and ALL^25^. Indeed, Notch signaling pathway inhibitors, such as gamma-secretase inhibitors, have been considered as promising tools for cancer therapy^24,26^. Additionally, Takam et al. described a modulatory effect of the Notch pathway in B-ALL, with Notch pathway suppression leading to increased chemosensitivity of B-ALL cells^27^. Autophagy was also previously associated with the development of ALL^17^. Collectively, these findings suggest that the Notch signaling and autophagy pathways contribute to the progression of B-ALL.

Based on the results of the proteomic analysis, ADAM17 and ATG3 were found to be enriched in the Notch and autophagy pathways, respectively. ADAM17 is a member of the ADAM family and is a membrane-related and structurally associated metalloproteinase^28^. A previous study showed that ADAM17 is responsible for the release of more than 70 membrane-tethered growth factors, cytokines, and cell surface receptors, including the Notch receptor, making it an important regulator of various biological processes, such as cell regeneration, apoptosis, and inflammation^29,30^. ADAM17 is the main protease for processing Notch and its upregulation contributes to the activation of the Notch signaling pathway, which in turn promotes tumorigenesis^31,32^. In contrast, Notch signaling pathway inhibition results in B-ALL cell apoptosis^27,33^. A study reported by Maria et al. has demonstrated that ADAM10 and ADAM17 were highly expressed in multiple T-ALL cells, and ADAM resulted in the activation of oncogenic Notch in T-cell lymphoblasts^34^. However, the expression and function of ADAM17 in B-ALL is barely investigated. ATG3 is a key regulator of autophagy, and its upregulation can lead to the activation of autophagy^35^. In turn, inhibition of autophagy reduced B-ALL cell survival and proliferation^17^. Evidences have found the high expression of ATG3 in various cancers, containing colon cancer^36^, hepatocellular carcinoma^37^, and acute myeloid leukemia^38^, which promoted the development of cancers through accelerating autophagy. Whereas the expression and function of ATG3 in B-ALL id rarely explored. In this study, the proteomic analysis demonstrated that both ADAM17 and ATG3 are highly expressed in patients with B-ALL, which was verified by western blot. Furthermore, receiver operating characteristic curves analyses confirmed that these proteins could effectively identify individuals with B-ALL from those without B-ALL, indicating that ADAM17 and ATG3 might be potential diagnostic biomarkers and therapeutic targets for B-ALL. However, the function of ADAM17 mediated Notch signaling pathway activation and ATG3 mediated autophagy in B-ALL need to be further investigated.

Moreover, our current study showed that several RNA splicing factors, including heterogeneous nuclear ribonucleoproteins C1/C2 (HNRNPCC), HNRNPU, HNRNPU-like protein 2, matrin-3 (MATR3), serine/arginine-rich splicing factor 6 (SRSF6), and RNA-binding protein Raly (RALY)^39^ were upregulated in patients with B-ALL. Alternative splicing of mRNA generates abundant, distinguishingly spliced RNA transcripts, which results in the spatial diversity of biological functions. As a hallmark of cancer, alternative splicing has been identified to be related to multiple oncological processes, including immune destruction, angiogenesis, metastasis, and cellular energetics^40^. Kuriyama et al. reported that knockdown of MATR3, which is highly expressed in malignant melanoma, leads to the suppression of proliferation and enhanced apoptosis of melanoma cells in vivo and in vitro^41^. Sun et al. showed that RALY is overexpressed in colorectal cancer and promotes cancer aggressiveness by regulating miRNA-mediated reprogramming of mitochondrial metabolism^42^. Numerous studies have demonstrated the carcinogenic effects of SRSF6 in multiple cancers, including colorectal cancer, lung cancer, ovarian cancer, and T-cell ALL^43^. However, whether the upregulation of exosome-loaded RNA splicing factors detected in our current study is involved in tumorigenesis and their potential functional mechanism in B-ALL needs to be clarified, which will be the focus of our follow-up studies.

In conclusion, this study revealed the proteomic profile of plasma-derived exosomes from patients with B-ALL and identified 342 DEPs. In addition, our findings suggest that overexpression of ADAM17 and ATG3 in plasma exosomes may contribute to the progression of B-ALL through the activation of the Notch signaling and autophagy pathways, respectively. Thus, these findings can pave the way for the development of novel diagnostic and therapeutic strategies for B-ALL.

## Methods

### Patients and sample preparation

A total of 18 newly diagnosed patients with B-ALL (median age: 34 years; range: 20–62 years) were recruited at the Second Xiangya Hospital of Central South University. Plasma samples from five of these patients were used for proteomic analysis, and all the 18 patients were used for further validation. The detailed baseline characteristics of these 18 patients enrolled in our current study are shown in Table 1. Plasma samples from 10 healthy individuals (median age: 31 years; range: 19–43 years) were used as controls, of which five were used for proteomics analysis and all of 10 healthy individuals were used for validation. Total blood samples from the patients and healthy individuals were collected in anticoagulant-coated tubes. The obtained blood samples were then centrifuged for 10 min at 2 000 × *g* at 4 °C for plasma isolation. Whole plasma samples were aliquoted and maintained at − 80 °C for subsequent experiments.

### Exosome isolation

Ultracentrifugation was performed to isolate the exosomes. Briefly, 6 mL of plasma from each patient and healthy individual was diluted five times with phosphate-buffered saline and centrifuged for 10 min at 300 × *g* at 4 °C. The obtained supernatant was centrifuged for 10 min at 2 000 × *g* at 4 °C. Thereafter, the supernatant was centrifuged for 30 min at 10 000 × *g* at 4 °C using an ultracentrifuge (Himac CP80WX; Hitachi, Tokyo, Japan), followed by centrifugation for 70 min at 120 000 × *g* at 4 °C. Finally, the exosome precipitate was collected, suspended in 300 μL phosphate-buffered saline, and stored at − 80 °C for subsequent analysis. The particle size distribution of the exosomes was determined by nanoparticle tracking analysis using a ZetaView apparatus (Particle Metrix, Meerbusch, Germany), and the data were analyzed using ZetaView software (Version 8.04.02).

### Transmission electron microscope observation

The collected exosomes were examined using a transmission electron microscope for morphology observation. For this purpose, 15 μL of plasma exosomes were loaded for 1 min on a copper grid and dried using filter paper. Thereafter, the absorbed plasma exosomes were stained for 1 min with 15 μL of 2% uranyl acetate at room temperature and dried using filter paper. The morphology of the exosomes was observed using an FEI Tecnai G2 Spirit transmission electron microscope (Thermo Fisher Scientific, Waltham, MA, USA).

### Quantitative proteomics analysis of exosome proteins and bioinformatics analysis

Liquid chromatography–electrospray ionization–tandem mass spectrometry was performed for proteomic analysis using an 1100 HPLC System equipped with a Zorbax Extend RP column (5 μm, 150 × 2.1 mm) (Agilent, Santa Clara, CA, USA), which was connected to a Q-Exactive mass spectrometer (Thermo Fisher Scientific) equipped with a Nanospray Flex source (Thermo Fisher Scientific). Mobile phase A consisted of 2% acetonitrile, and mobile phase B consisted of 98% acetonitrile. The primary mass spectrometry scans were set to a range of 300–1600 m/z with a resolution of 70,000. The secondary scanning resolution was set to 17,500. The raw data were analyzed using Proteome Discover software (version 2.3, Thermo Fisher Scientific). The false discovery rate was set at 0.01. Trusted proteins were screened according to the criteria of Score Sequest HT > 0 and unique peptides ≥ 1. Differentially expressed exosome proteins were identified based on the criteria of ≥ twofold change and *p* < 0.05 (*t*-test). GO and KEGG database (https://www.genome.jp/kegg/)^44–46^ were used for functional and pathway enrichment analysis of differentially expressed exosome proteins, respectively, using Fisher’s exact test. Statistical significance was set at *p* < 0.05.

### Western blot analysis

Western blot was performed to identify exosomes and verify the predicted proteins associated with B-ALL progression. Whole exosomes in each sample were lysed in radioimmunoprecipitation assay lysis buffer and quantified using a bicinchoninic acid protein assay kit (23,227; Thermo Fisher Scientific). Thereafter, 20 μg of protein was separated by 12% sodium dodecyl sulfate–polyacrylamide gel electrophoresis and transferred onto a polyvinylidene fluoride membrane (Millipore, Burlington, MA, USA). The membranes were blocked with 5% skim milk and incubated with primary antibodies supplied by Abcam (Cambridge, UK) against TSG101 (ab125011), CD81 (ab109201), calnexin (ab133615), ATG3 (ab108251), and ADAM17 (ab28233) overnight at 4 °C. The membranes were then incubated for 1 h with a secondary antibody at room temperature. A commercial chemiluminescence kit was used to visualize the immunoreactive blots, which were photographed using a Tanon 5200 camera (Tanon, Shanghai, China).

### Statistical analysis

Exosomal protein levels in patients with B-ALL and healthy individuals were compared using the Mann–Whitney *U*-test for non-parametric analysis. Statistical significance was defined as a two-sided *p*-value < 0.05. Receiver operating characteristic curves were generated for individual exosomal proteins to evaluate their clinical utility. All data analyses were performed using the GraphPad Prism 7 (GraphPad Software, San Diego, CA, USA).

### Ethics statement

This study was approved by the Ethics Committee of the Second Xiangya Hospital of Central South University, and informed consent was obtained from all the patients. The study was performed in accordance with the Declaration of Helsinki.

## Supplementary Information

Supplementary Information 1.

Supplementary Information 2.

Supplementary Information 3.

Supplementary Information 4.

Supplementary Information 5.

Supplementary Information 6.

Supplementary Information 7.

Supplementary Information 8.

## Data Availability

The data that support the findings of this study are available from the corresponding author upon reasonable request.
