# Supplementary material for: Proteomic profiling of plasma exosomes from patients with B-cell acute lymphoblastic leukemia
Source: Sci Rep. 2022 Jul 13;12:11975. doi: 10.1038/s41598-022-16282-4 (PMC9279438; doi:10.1038/s41598-022-16282-4)
Supplement: Supplementary file 1 — Supplementary Information 1. [file 41598_2022_16282_MOESM1_ESM.pdf]

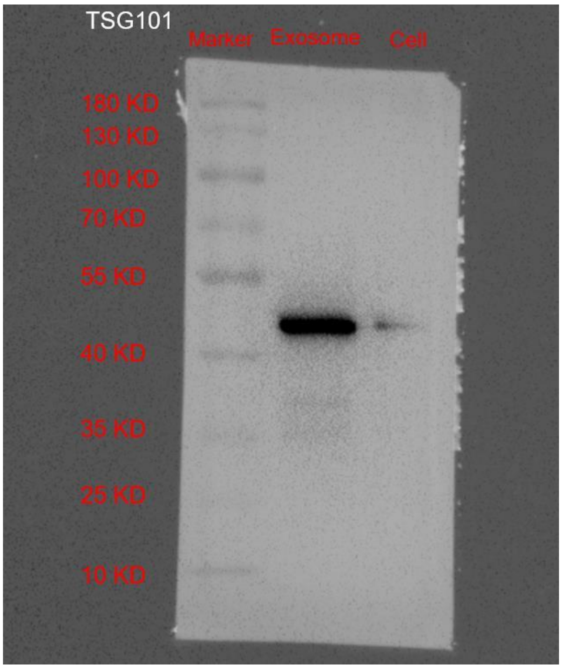

Figure S1 Original western blot for TSG101 of B-ALL patient

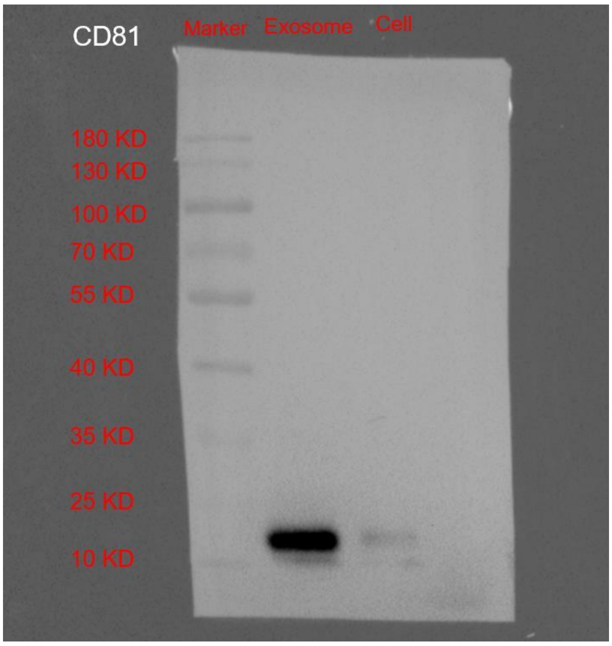

Figure S2 Original western blot for CD81 of B-ALL patient

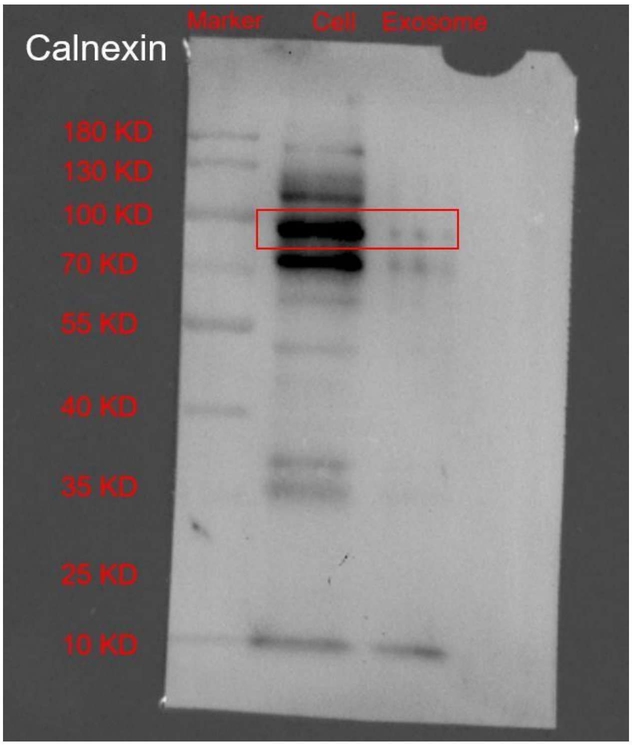

Figure S3 Original western blot for calnexin of B-ALL patient

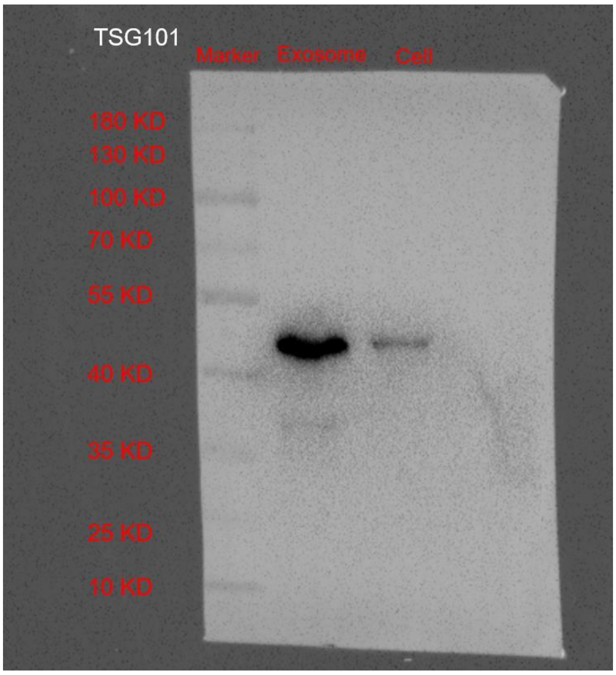

Figure S4 Original western blot for TSG101 of healthy control

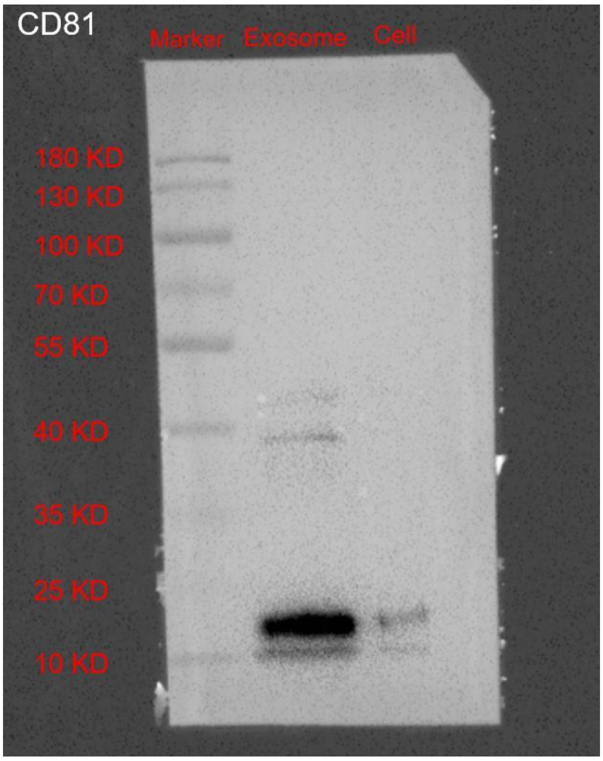

Figure S5 Original western blot for CD81 of healthy control

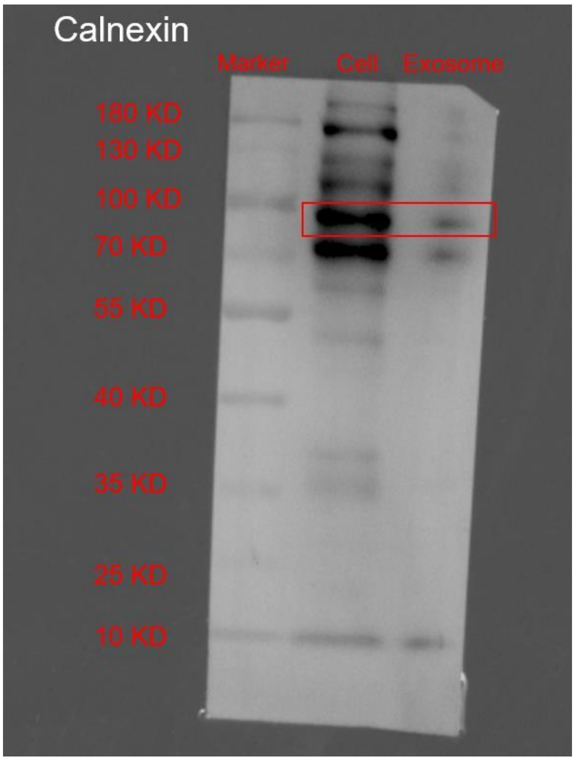

Figure S6 Original western blot for calnexin of healthy control

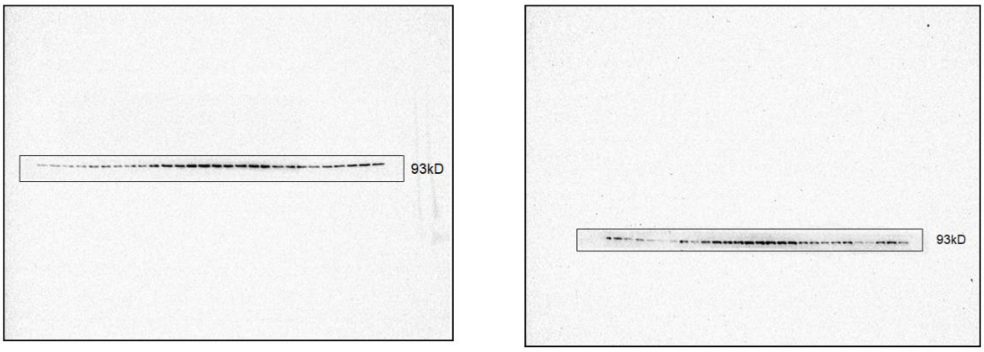

Figure S7 Original and repetitive western blot for ADAM17 in Figure 6 (The blots were cut prior to hybridization with antibodies)

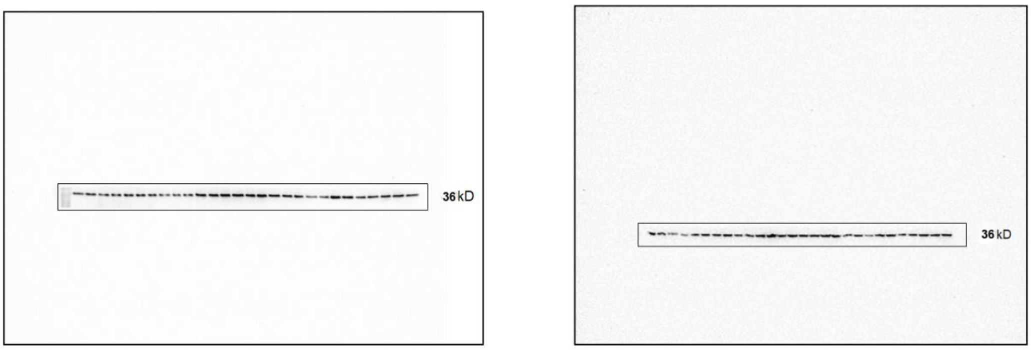

Figure S8 Original and repetitive western blot for ATG3 in Figure 6 (The blots were cut prior to hybridization with antibodies)

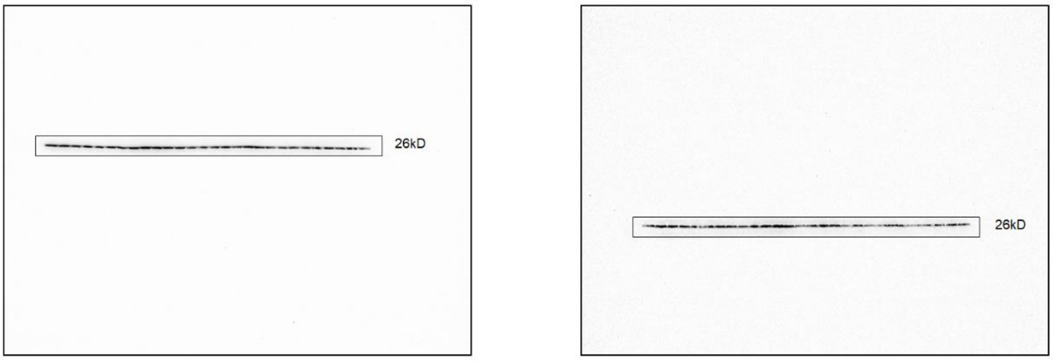

Figure S9 Original and repetitive western blot for CD81 in Figure 6 (The blots were cut prior to hybridization with antibodies)
